# Model-based deep learning with fully connected neural networks for accelerated magnetic resonance parameter mapping

**DOI:** 10.1007/s11548-025-03356-7

**Published:** 2025-05-03

**Authors:** Naoto Fujita, Suguru Yokosawa, Toru Shirai, Yasuhiko Terada

**Affiliations:** 1 Institute of Pure and Applied Physics, University of Tsukuba, Tsukuba, Japan; 2 FUJIFILM Corporation, Medical Systems Research and Development Center, Minato-ku, Tokyo, Japan

**Keywords:** Deep neural networks, MRI reconstruction, Quantitative MRI

## Abstract

**Purpose:**

Quantitative magnetic resonance imaging (qMRI) enables imaging of physical parameters related to the nuclear spin of protons in tissue, and is poised to revolutionize clinical research. However, improving the accuracy and clinical relevance of qMRI is essential for its practical implementation. This requires significantly reducing the currently lengthy acquisition times to enable clinical examinations and provide an environment where clinical accuracy and reliability can be verified. Deep learning (DL) has shown promise in significantly reducing imaging time and improving image quality in recent years. This study introduces a novel approach, quantitative deep cascade of convolutional network (qDC-CNN), as a framework for accelerated quantitative parameter mapping, offering a potential solution to this challenge. This work aims to verify that the proposed model outperforms the competing methods.

**Methods:**

The proposed qDC-CNN is an integrated deep-learning framework combining an unrolled image reconstruction network and a fully connected neural network for parameter estimation. Training and testing utilized simulated multi-slice multi-echo (MSME) datasets generated from the BrainWeb database. The reconstruction error with ground truth was evaluated using normalized root mean squared error (NRMSE) and compared with conventional DL-based methods. Two validation experiments were performed: (Experiment 1) assessment of acceleration factor (AF) dependency (AF = 5, 10, 20) with fixed 16 echoes, and (Experiment 2) evaluation of the impact of reducing contrast images (16, 8, 4 images).

**Results:**

In most cases, the NRMSE values of S0 and T2 estimated from the proposed qDC-CNN were within 10%. In particular, the NRMSE values of T2 were much smaller than those of the conventional methods.

**Conclusions:**

The proposed model had significantly smaller reconstruction errors than the conventional models. The proposed method can be applied to other qMRI sequences and has the flexibility to replace the image reconstruction module to improve performance.

**Supplementary Information:**

The online version contains supplementary material available at 10.1007/s11548-025-03356-7.

## Introduction

Quantitative magnetic resonance imaging (qMRI) enables imaging of physical parameters related to the nuclear spin of protons in tissue (e.g., proton density, relaxation time, diffusion coefficient) [1, 2]. Conventional weighted images are qualitative information reflecting a complex set of quantitative values, and diagnosis is based on detecting morphological defects and contrast changes in these images. If quantitative parameter information can be incorporated into the diagnosis in addition to this information, a more objective diagnosis will be possible [3]. In addition, some research uses quantitative information for data analysis and diagnosis. These include cancer staging [4], automated diagnosis through machine learning [5], detection of microstructural abnormalities in the brain [6], and diagnosis of cartilage lesions in the knee [7].

However, improving the accuracy and clinical relevance of qMRI remains a major challenge for its practical implementation. This improvement is not straightforward partially because qMRI has only been used in limited clinical practice because of the long acquisition time [3, 8, 9]. In qMRI, multiple contrast images are required for estimating quantitative maps, which has prompted extensive research on accelerated imaging techniques. Two main approaches have emerged to address this challenge: the development of pulse sequences [10] and undersampling (US) strategies represented by compressed sensing (CS) [11], parallel imaging (PI) [12–14], and row-rank methods [15].

It is common practice to divide the process into two steps to obtain parameter maps: image reconstruction from US data and parameter fitting with a signal model. In recent years, advances in deep learning (DL) have made it possible to replace one or both of these processing steps, achieving significant reductions in acquisition time while maintaining image quality [16–19].

The state-of-the-art DL-based image reconstruction uses the unrolling approach, which replaces a regularization term used in traditional iterative CS algorithms with deep neural networks (DNNs) and unrolling them with iterative algorithms. For example, the deep cascade of convolutional neural networks (DC-CNN) [18] adopts a DL-based L2 norm as the regularization term and is constructed by unrolling it with a closed-form solution. Model-based deep learning (MoDL) [17] uses the same regularization term and unrolls it using a conjugate gradient method. Pixel-wise quantitative parameter estimation using fully connected neural networks (FCNNs) is also actively studied in various applications. Several studies have demonstrated the advantages of FCNNs: improved accuracy and noise robustness compared to conventional methods [20], more stable estimation than least square fitting (LSF) [21], and maintained image quality even with the reduced number of contrast images [22].

Several studies have reported end-to-end (E2E) DNNs that integrate image reconstruction and quantitative parameter estimation. Liu et al. [23] proposed U-Net-based parameter mapping methods called the model-augmented neural network with incoherent k-space sampling (MANTIS) framework. MANTIS takes US k-space data from multiple echoes as input and directly maps them to parameter images. They also introduced a data consistency loss incorporating a signal model. Li et al. [24] proposed SUPERMAP, an improved vision of MANTIS. SUPERMAP reduces the number of contrast images required for quantitative parameter estimation by modifying the input/output images to be patch-wise and the data consistency loss to be calculated per patch. One limitation with these direct mapping approaches is that they may have lower reconstruction performance since they did not use unrolled networks.

To address this, Yun et al. [25] proposed a deep model-based magnetic resonance parameter mapping network using an unrolled network (DOPAMINE) and showed that it effectively improves the network performance. DOMAPINE directly incorporates a signal model into an unrolled DL model; it consists of two network parts: the first network estimates parameter images from zero-filled (ZF) input images, and the subsequent network performs denoising using these parameter images as input while maintaining data consistency with the signal model. However, DOPAMINE requires the differential form of the signal model to be analytically incorporated within the network, making it impossible to apply to complicated signal models that do not have analytical solutions and limiting its applicability to various qMRI sequences.

These DL-based qMRI methods have limitations, as described above, and there is much room for improvement. One possible solution is to combine an image reconstruction module using an unrolled network with a parameter-fitting network using FCNN, and connect them by a data consistency loss using a signal model. Based on this idea, we propose quantitative DC-CNN (qDC-CNN), a framework that uses a “reconstruction module” and a “mapping module,” which are used for image reconstruction and quantitative parameter estimation, respectively. The reconstruction module uses DC-CNN, one of the state-of-the-art models, and the mapping module uses FCNN. Similar to MANTIS, the proposed network uses data consistency loss incorporating the signal model, so that any signal model can be easily incorporated, even without an analytical solution. In addition, as with DOPAMINE, the proposed network uses an unrolled network, so that high reconstruction performance is expected. The objective of this work is to verify that the proposed model outperforms the competing methods. Specifically, we measure the reconstruction error of reconstructed S0 and T2 images using the normalized root mean squared error (NRMSE) over simulated brain MRI data. We show that the proposed model exhibits smaller NRMSE values than the competing methods.

## Material and methods

### Study workflow

**Fig. 1:**
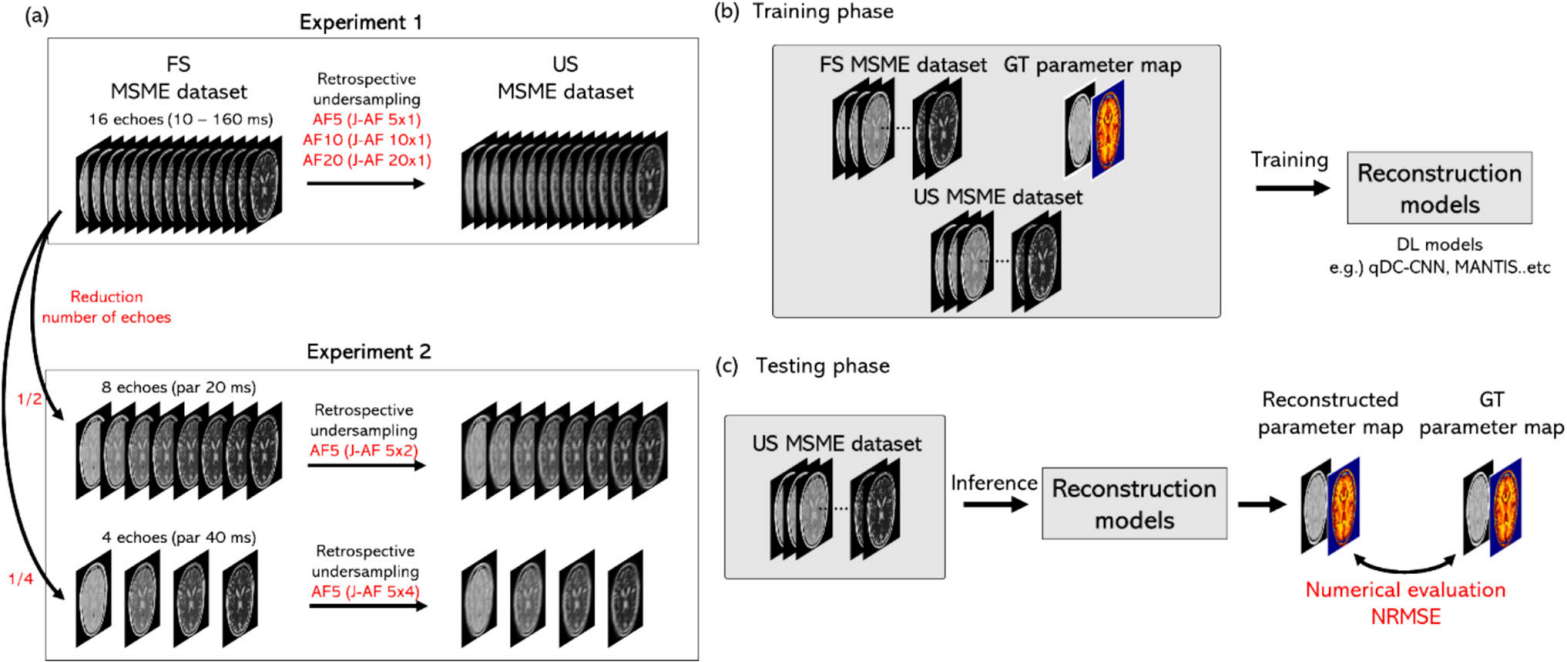
Workflow of this study. AF: acceleration factor, FS: fully sampled, US: undersampled, MSME: multi-slice multi-echo, GT: ground truth, NRMSE: normalized root mean squared error

Figure 1 shows the workflow of this study. The proposed DL network outputs quantitative maps from multiple contrast (multi-contrast) images. The subject of this study is T2 mapping with multi-slice multi-echo (MSME) sequences. We chose MSME because it is a standard method for T2 mapping and has been extensively used in previous studies [23, 26, 27]. Experiment 1 focused on increasing the acceleration factor (AF), and Experiment 2 focused on reducing the number of acquired multi-contrast images (the number of multi-echo signals) (Fig. 1a).

In the training phase, three DNNs were trained using the training dataset. Fully sampled (FS) data, US data, and ground truth (GT) quantitative maps were used to train the DL models (Fig. 1b). In the testing phase, the quantitative maps obtained using the test dataset were evaluated with normalized root mean squared error (NRMSE) for each reconstruction method (Fig. 1c).

### Problem formulation for accelerated quantitative parameter mapping

In quantitative MRI, multi-contrast images are acquired. In this section, we describe the process of sampling and encoding multi-contrast images. When the MSME sequence is targeted, multi-contrast images are obtained from the multi-echo images. To formulate such a sampling process, we define the encoding operator ${\varvec{E}}_{{{\varvec{\Omega}}}} = \left[ {{\varvec{M}}_{{{\varvec{\varOmega}}_{1} }} {\varvec{F}},\user2{ M}_{{{\varvec{\varOmega}}_{2} }} {\varvec{F}}, \ldots ,{\varvec{M}}_{{{\varvec{\varOmega}}_{{\varvec{P}}} }} {\varvec{F}}} \right]^{T} \!:\!{\mathbb{R}}^{P\! \times\! H \!\times\! W \!\times \!2} \mapsto {\mathbb{R}}^{P \!\times\! H \!\times \!W \!\times \!2}$ that encodes multi-contrast (multi-echo) images together, ${\varvec{F}}$ is the discrete Fourier operator, and ${\varvec{M}}_{{\varvec{X}}}$ is the sampling operator that fills unmeasured points $\overline{X}$ (regions other than $X$) in k-space with 0. P is the number of input echoes, ${\Omega }_{{\text{i}}}$ is the sampling region on k-space corresponding to the i-th contrast image, and ${\Omega } = \left\{ {\Omega_{1} ,\Omega_{2} , \ldots ,\Omega_{P} } \right\}$ is the set of these sampling regions.

The CS image reconstruction problem for multi-contrast images can be formulated using this sampling operator as follows:

1 $$ \begin{array}{*{20}c}    {x_{{{\text{rec}}}}  = \mathop {{\text{argmin}}}\limits_{x} \left( {R\left( x \right) + \lambda \left\| {\varvec{E}_{{\mathbf{\Omega }}} x - y_{\mathbf{\Omega }}} \right\|_{{{{\Omega }}\;2}}^{2} } \right),}  \\   \end{array}  $$

where $x \in {\mathbb{R}}^{P \times H \times W \times 2}$ is the multi-contrast image, $x_{{{\text{rec}}}}$ is the final reconstructed image, $\left\| {\varvec{E}_{{\mathbf{\Omega }}} x - y_{{{\Omega }}} } \right\|$ is the data consistency term, and $R\left( x \right)$ is the regularization term. $\lambda$ is the parameter that balances the data consistency term and the regularization term. As a reference, we also calculated the ZF reconstruction image $x_{zf} = {\varvec{E}}_{{\varvec{\varOmega}}}^{{\varvec{H}}} y_{{\Omega }}$.

The MSME signal model is expressed as follows:

2 $$ \begin{array}{*{20}c} {S\left( {S_{0} ,T_{2} ,{\text{TE}}} \right) = S_{0} \exp \left( { - \frac{{{\text{TE}}}}{{T_{2} }}} \right),} \\ \end{array} $$

where $S_{0}$ is a constant proportional to proton density, $S\left( {S_{0} ,T_{2} ,{\text{TE}}} \right)$ is the signal model, and ${\text{TE}}$ is echo time.

### Proposed network

#### Overview of the proposed network

qDC-CNN is an E2E framework that performs all steps from image reconstruction to quantitative parameter estimation. This framework has a structure that connects an image reconstruction module, $f_{{{\text{rec}}}}$, and a parameter mapping module, $f_{{{\text{map}}}}$. $f_{{{\text{rec}}}}$ takes US multi-contrast images as inputs and outputs reconstructed multi-contrast images, $x_{{{\text{rec}}}}$. $f_{{{\text{map}}}}$ performs the conversion from $x_{rec}$ to the quantitative parameter maps, $p_{{{\text{rec}}}} = \left( {T_{2} ,{\text{Re}} \left\{ {S_{0} } \right\},{\text{Im}} \left\{ {S_{0} } \right\}} \right) \in {\mathbb{R}}^{3 \times H \times W}$. Here, $S_{0} = {\text{Re}} \left\{ {S_{0} } \right\} + j{\text{Im}} \left\{ {S_{0} } \right\}$. Based on this, the input/output of $f_{{{\text{rec}}}}$ and $f_{{{\text{map}}}}$ is formulated as follows:

3 $$ \begin{array}{*{20}c} {x_{{{\text{rec}}}} = f_{{{\text{rec}}}} \left( {E_{{\Omega }}^{H} y_{{\Omega }} } \right)} \\ \end{array} $$

4 $$ \begin{array}{*{20}c} {p_{{{\text{rec}}}} = f_{{{\text{map}}}} \left( {x_{{{\text{rec}}}} } \right)} \\ \end{array} $$

Here, $y_{{\Omega }} \in {\mathbb{R}}^{P \times H \times W \times 2}$ is the US multi-contrast k-space data. In this study, the reconstruction module was constructed by unrolling with the closed-form solution proposed by Schlemper et al. [18]. The details of the pretraining are described in Supplementary Information A. The mapping module $f_{{{\text{map}}}}$ has been pretrained to increase the stability of the E2E training. The details of the pretraining are described in the Supplementary Information A2.

#### Training strategy

**Fig. 2:**
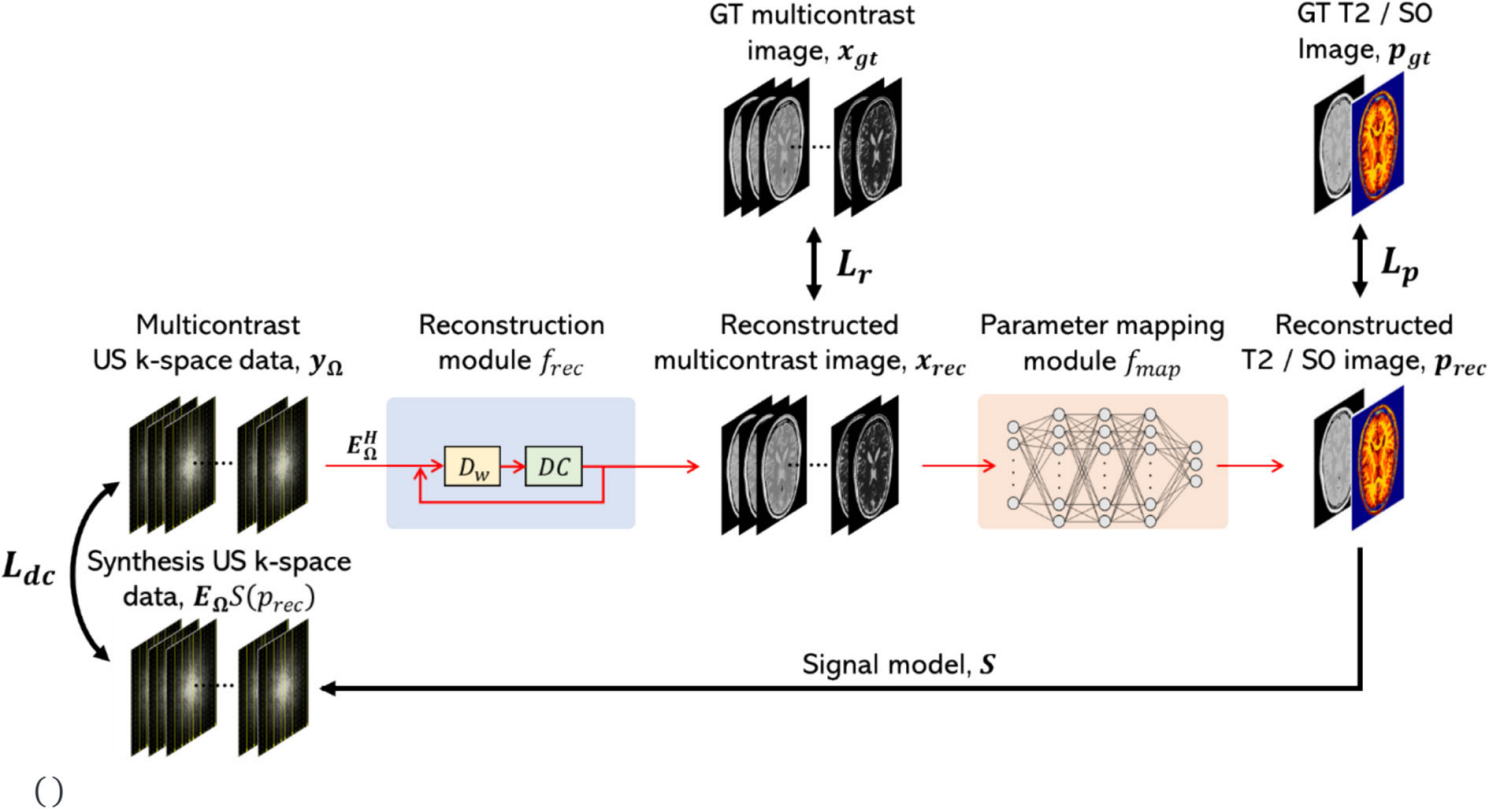
Loss function of this study. US: undersampled, GT: ground truth. DC is data consistency and Dw is denoiser. See Figure S1 for details

We trained each network with three losses. $L_{r} = \left\| {x_{{{\text{gt}}}} - x_{\text{rec}}} \right\|^{{2}}_{2}$ evaluated the pixel-wise error between the GT multi-contrast images $x_{{{\text{gt}}}}$ and reconstructed multi-contrast images $x_{{{\text{rec}}}}$, $L_{p} = \left\| {p_{{{\text{gt}}}} - p_{\text{rec}}} \right\|^{2}_{2}$ evaluates the error between the GT quantitative maps $p_{{{\text{gt}}}}$ and the reconstructed quantitative maps $p_{{{\text{rec}}}}$, and $L_{{{\text{dc}}}} = \left\| {y_{{\Omega }} - {\varvec{E}}_{{{\varvec{\Omega}}}} S\left( {p_{{{\text{rec}}}} } \right)} \right\|_{2}^{2}$ evaluates the error (data consistency) between the measured k-space data and the k-space data estimated from the reconstructed quantitative maps (Fig. 2). Here, $S\left( {p_{{{\text{rec}}}} } \right)$ are multi-contrast images calculated from reconstructed quantitative maps with the signal model. The total loss was $L_{{{\text{total}}}} = \lambda_{r} L_{r} + \lambda_{p} L_{p} + \lambda_{{{\text{dc}}}} L_{{{\text{dc}}}}$. Here, $\lambda_{r} ,\lambda_{p} ,\lambda_{{{\text{dc}}}}$ are balanced parameter in each loss. Note that they are introduced to adjust the absolute value of each loss and balance it with the other loss components and are not scaled in the range of 0 to 1. In this study, $\lambda_{r} = 1,\lambda_{p} = 1.0 \times 10^{ - 2} ,\lambda_{{{\text{dc}}}} = 1.0 \times 10^{ - 6} .$

### Network evaluation

#### Dataset

**Fig. 3:**
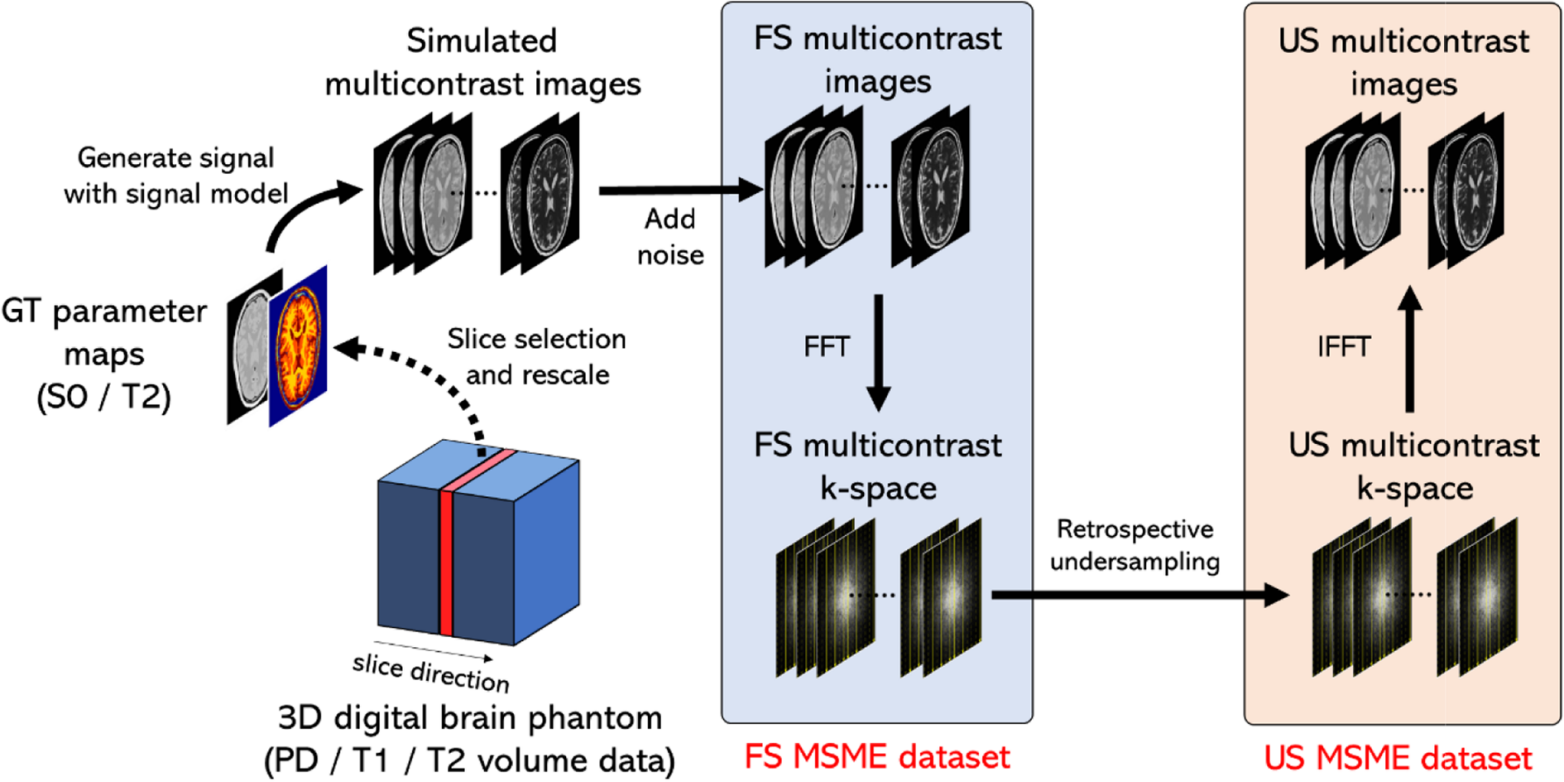
Procedures for creating a simulation dataset. FS: fully sampled, US: undersampled, MSME: multi-slice multi-echo, GT: ground truth

The procedure for creating the dataset for this study is shown in Fig. 3. GT datasets were prepared using brain digital phantoms created from 20 healthy adults. The brain digital phantoms were obtained from the BrainWeb project [28] (https://brainweb.bic.mni.mcgill.ca/). We created MSME images by simulation. The 20 cases were split into 16, 2, and 2 cases for training, validation, and testing, respectively. The field of view of the digital phantom images was 220 mm × 220 mm × 181 mm, and the matrix size was 362 × 362 × 434. From this dataset, axial cross-sectional images were selected from the center to have a slice thickness of 2 mm, a slice gap of zero, and the number of slices was 60. Next, $S_{0}$ images were created according to $S_{0} = {\text{PD}}\left( {1 - \exp \left( { - {\text{TR}}/T_{1} } \right)} \right)$, and then scaled so that the matrix size was 256 × 256. The $S_{0}$ image was also normalized so that the pixel values ranged from 0 to 1. FS multi-contrast images were created as TE/TR = [10, 20, 30, …, 160]/7000 ms based on the signal model in Eq. (2). The FS multi-contrast k-space data was created by performing the Fourier transform of the FS multi-contrast images. Complex Gaussian noise was added to the k-space data. The noise was adjusted to a peak signal-to-noise ratio (PSNR) of 40 dB at TE = 10 ms compared to before the noise addition. US multi-contrast k-space data were created by retrospectively undersampling the FS multi-contrast k-space data. US multi-contrast images were created from the US multi-contrast k-space data by inverse Fourier transform.

#### Compared models

We compared six reconstruction methods (A)–(F) for qMRI (Table 1). The methods (A)–(D) used DNNs. The method (B) is the baseline DNN for qMRI, where $L_{{{\text{dc}}}}$ and $L_{p}$ were used as loss terms. The U-Net in (B) was implemented using the public code in the fastMRI project (https://github.com/facebookresearch/fastMRI), and the number of input/output channels was the same as in (A). We used the models (C) and (D) to investigate the contribution of $f_{{{\text{map}}}}$ to the reconstruction performance. The model (C) was trained using only $L_{r}$ as the loss function. In the models (C) and (D), LSF was used instead of $f_{{{\text{map}}}}$. The method (E) used k-t SLR [15] instead of $f_{{\text{ rec}}}$, which is the baseline for non-DL image reconstruction. We used the MATLAB source code for k-t SLR (https://research.engineering.uiowa.edu/cbig/content/matlab-codes-k-t-slr). The hyperparameters for k-t SLR were tuned by grid search [29] using the validation dataset. The model (F) was the ZF reconstruction as described in 2.2. In methods (E) and (F), quantitative parameter estimation was performed using LSF.

**Table 1:** List of models used in this study. ZF: zero-filled reconstruction. FCNN: fully connected neural network. LSF: least squared fitting. E2E: end-to-end

Method	DL-type	Image reconstruction	Parameter estimation	Training method (loss function)
(A) qDC-CNN	E2E	DL-based unrolled network (DC-CNN)	Pixel-wise DL (FCNN)	E2E training ($L_{r} , L_{p} , L_{{{\text{dc}}}}$)
(B) MANTIS	E2E	–	Image-to-Image (U-Net)	E2E training ($L_{p} , L_{dc}$)
(C) DC-CNN	Image reconstruction only	DL-based unrolled network (DC-CNN)	Pixel-wise non-DL (LSF)	Image space training ($L_{r}$)
(D) qDC-CNN w/o fmap	Image reconstruction only	DL-based unrolled network (DC-CNN)	Pixel-wise non-DL (LSF)	E2E training ($L_{r} , L_{p} , L_{{{\text{dc}}}}$)
(E) k-tSLR	Non-DL	Conventional non-DL	Pixel-wise non-DL (LSF)	–
(F) ZF	Non-DL	Conventional non-DL	Pixel-wise non-DL (LSF)	–

The learning rate for all networks was $1.0 \times 10^{ - 4}$ and Adam was used as the optimizer. Early stopping was used for training. The training was terminated when the loss of validation data did not improve by more than 10 epochs. In training, the sampling region was changed for each batch, as suggested by Liu et al. [23].

#### Experiments

Experiments 1 and 2 were performed to verify the proposed method to reduce data acquisition from two aspects: increasing AF and reducing the number of multi-contrast images. Experiment 1 compared methods (A)–(F) under AF = 5, 10, and 20, with the number of multi-contrast images fixed at 16. In Experiment 2, we compared models (A) and (B) when AF was fixed at 5, and the number of multi-contrast images was changed to 4, 8, and 16. See Supplementary Information B2 and B3 about the learning environment and sampling patterns. In this study, preliminary experiments were conducted to investigate the influence of the parameters $\lambda_{p} ,\lambda_{{{\text{dc}}}}$. The results are presented in Supplementary Information C.

#### Metrics

To evaluate parameter maps, we calculated the NRMSE as follows:

5 $$ \begin{array}{*{20}c} {{\text{NRMSE}}\left( {x,y} \right) = \frac{{\left\| {x - y} \right\|_{2,\Phi } }}{{\left\| x \right\|_{2,\Phi } }}} \\ \end{array} $$

Here, x and y are the GT and output images, respectively, and $\left\| \cdot \right\|_{2,\Phi }$ is the L2 norm of signal values in the region $\Phi$ in the image. To evaluate contrast images, we calculated the peak signal-to-noise ratio (PSNR), defined as, ${\text{PSNR}} = 10\log_{10} \left( {L^{2} /\left\| {x - y} \right\|_{2,\Phi } } \right)$, where $L$ is the maximum intensity.

## Results

### Dependence on AF (Experiment 1)

**Fig. 4:**
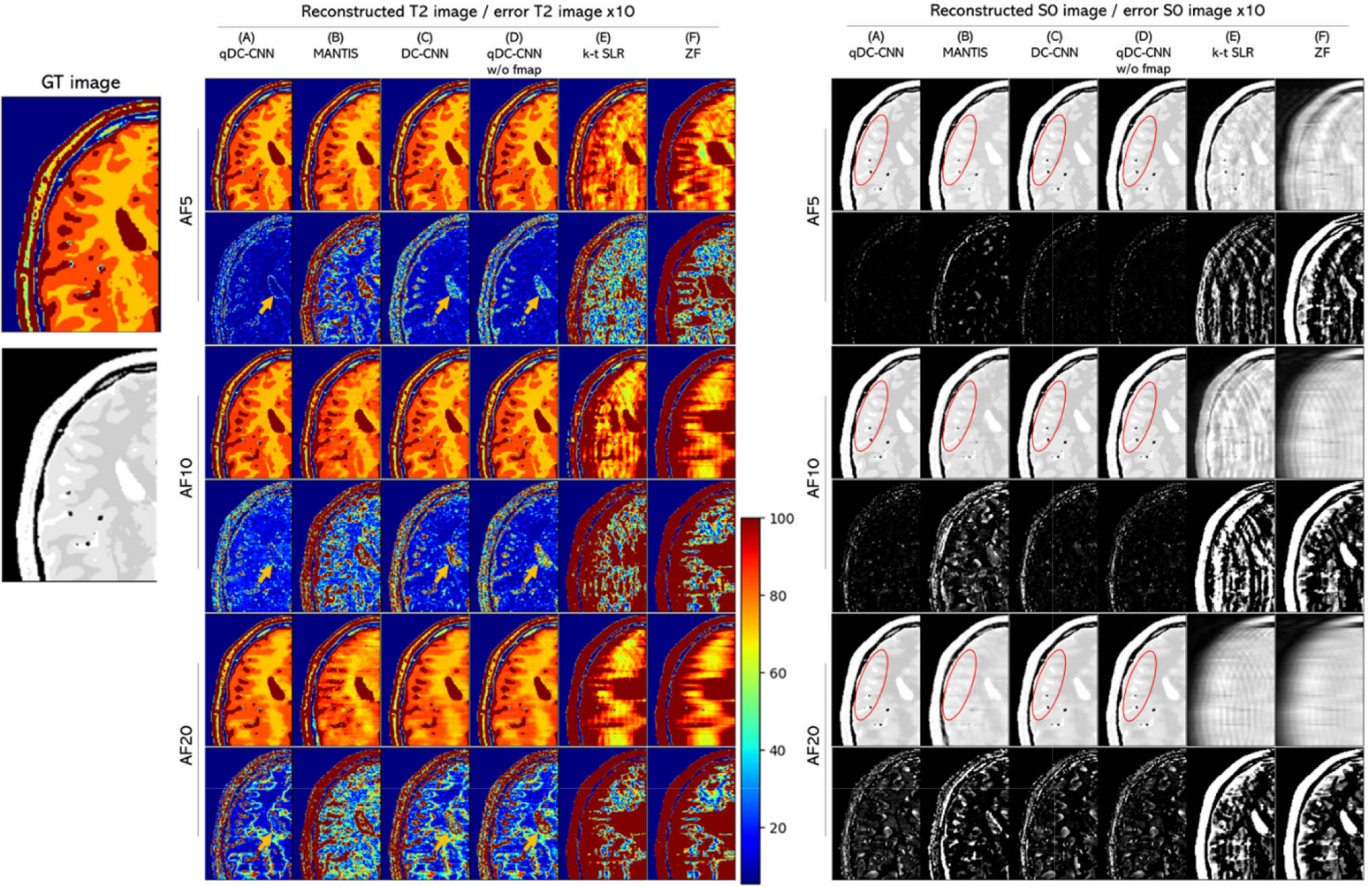
Reconstructed T2 and S0 maps and error images for different AFs (Experiment 1). The first row of each AF represents the reconstructed image and the second row represents the error image. Error images are emphasized by 10 times. The unit for the color bar is milliseconds. GT: ground truth, AF: acceleration factor

Figure 4 shows the T2 and S0 parameter maps reconstructed with methods (A)–(F) for different AFs. For AF = 5, the non-DL method, k-t SLR (E), showed significant artifacts, while the DL-based methods (A)–(D) showed highly accurate reconstruction performance with few artifacts. However, when comparing the images for AF = 10, MANTIS (B) tended to show blurred contours in the T2 and S0 maps and a noticeable loss of anatomical structures because of excessive smoothing, which was not the case for qDC-CNN (A), DC-CNN (C), and qDC-CNN w/o fmap (D). In the S0 maps, the boundary between white matter and gray matter (marked in red in Fig. 4) was delineated in qDC-CNN (A), DC-CNN (C), and qDC-CNN w/o fmap (D) at AF = 20 but not in MANTIS (B). qDC-CNN (A) showed fewer T2 errors than DC-CNN (C) and qDC-CNN w/o fmap (D) in the cerebrospinal fluid region (as indicated by yellow arrows in the figure).

Table 2 shows the mean and standard deviation of the NRMSE of the T2 and S0 maps. qDC-CNN (A) showed the smallest NRMSE values, indicating the highest performance among the five models. Furthermore, qDC-CNN (A) had approximately one-quarter of the S0 and T2 NRMSE values compared to MANTIS (B). The NRMSE increased with AF for all methods and was large for AF = 20. qDC-CNN w/o fmap (D) showed smaller NRMSE values and performed better than the DC-CNN (C). Since qDC-CNN w/o fmap (D) used data consistency and parameter mapping losses and DC-CNN (C) did not, these two factors likely had a positive effect on performance. qDC-CNN (A) using fmap outperformed qDC-CNN w/o fmap (D) using LSF, especially in estimating T2. This shows the advantage of using fmap instead of LSF.

**Table 2:** NRMSE of T2 and S0 maps estimated from each reconstruction method in Experiment 1. The smallest NRMSE values among the models (A)-(E) are shown in bold. AF: acceleration factor

Parameter	AF	(A) qDC-CNN	(B) MANTIS	(C) DC-CNN	(D) qDC-CNN w/o fmap	(E) k-tSLR	(F) ZF
S0	5	**1.63** ± 0.27	6.88 ± 0.36	2.10 ± 0.29	1.79 ± 0.23	6.41 ± 0.21	25.15 ± 0.76
	10	**2.69** ± 0.14	10.57 ± 0.63	3.21 ± 0.17	2.78 ± 0.13	22.56 ± 0.56	32.00 ± 0.80
	20	**6.59** ± 0.65	19.44 ± 0.90	7.76 ± 0.69	6.70 ± 0.66	31.46 ± 0.83	32.96 ± 0.82
T2	5	**2.25** ± 0.41	11.61 ± 0.63	10.18 ± 1.16	9.61 ± 1.20	15.15 ± 0.86	40.83 ± 0.80
	10	**3.62** ± 0.16	18.15 ± 0.98	9.47 ± 0.87	8.37 ± 0.94	43.30 ± 2.27	48.08 ± 0.93
	20	**11.58** ± 0.84	41.34 ± 1.58	17.26 ± 1.19	14.38 ± 1.11	48.75 ± 0.97	50.16 ± 0.90

We also conducted a numerical evaluation of each contrast image, except for MANTIS (B), which did not directly output the contrast images. See Supplementary Table S1 for these results. Similar to the S0 and T2 map results, the proposed method showed the highest PSNR values.

### Dependence on the number of multi-contrast images (Experiment 2)

**Fig. 5:**
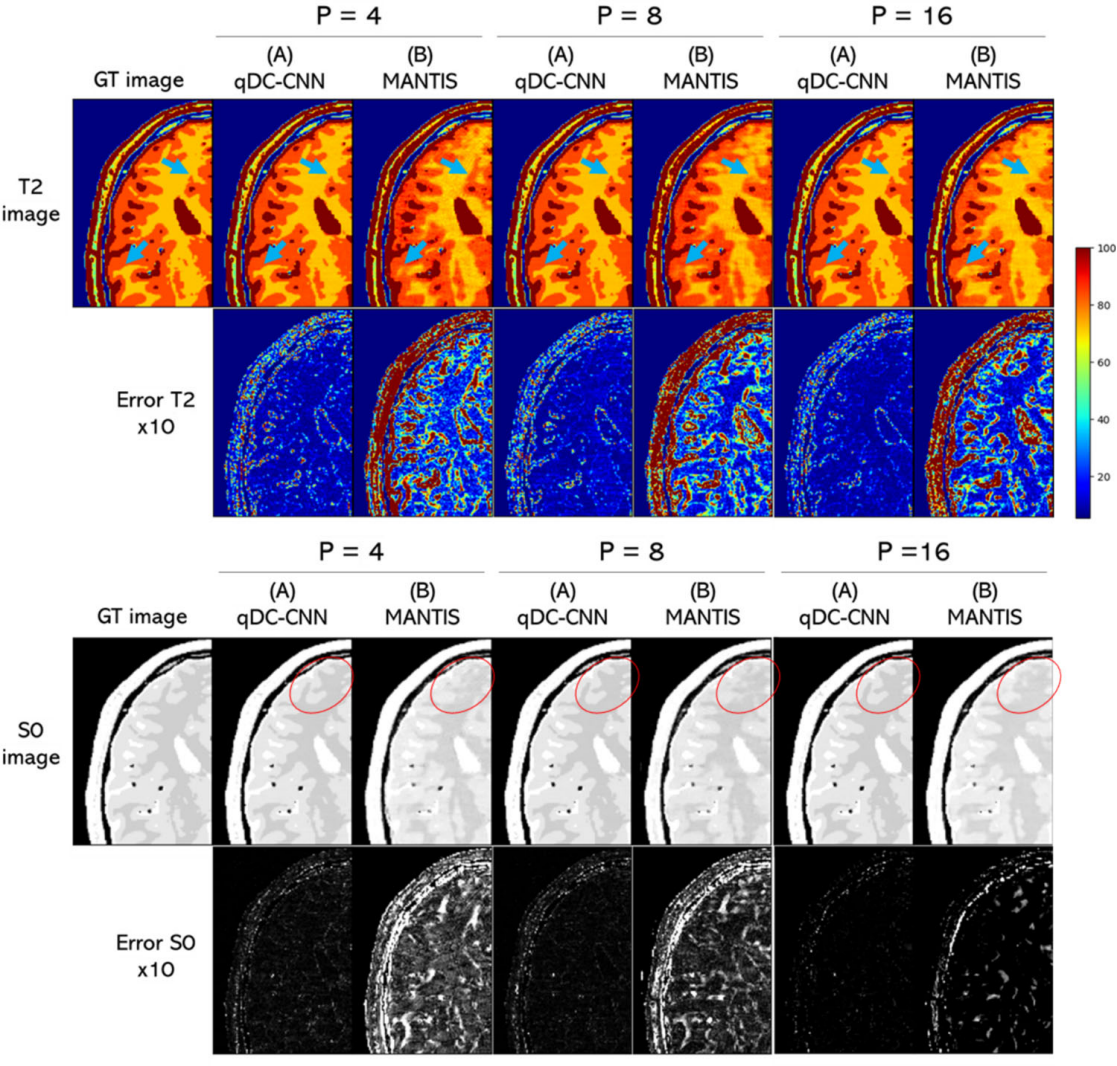
Reconstructed T2 and S0 maps and error images for different numbers of multi-contrast images (Experiment 2). The results with the models (A) and (B) were shown. P is the number of contrast images. Error images are emphasized by 10 times. The unit for the color bar is ms. GT: ground truth, AF: acceleration factor

Figure 5 shows the T2 and S0 maps reconstructed with qDC-CNN (A) and MANTIS (B) when the number of multi-contrast images was changed to 4, 8, and 16. qDC-CNN (A) showed high-quality T2 maps for all cases (the blue arrows in the T2 maps) and had small errors regardless of the number of multi-contrast images. In the T2 maps reconstructed with MANTIS (B), the structure and boundary between white and gray matter became ambiguous, or nonexistent structures appeared (light blue arrows), especially for the case with a small number of multi-contrast images. The same trend was observed in the S0 maps (red circles in the figure).

Table 3 shows the mean and standard deviation of the NRMSE of the S0 and T2 maps. qDC-CNN (A) showed smaller NRMSE values than MANTIS (B). We also conducted a numerical evaluation of each contrast image in Experiment 2. See Supplementary Table S2 for these results. Similar to the S0 and T2 map results, the PSNR values remained high regardless of the number of contrasts.

**Table 3:** NRMSE of T2 and S0 maps estimated from each reconstruction method in Experiment 2. The smallest NRMSE values among the models (A) and (B) are shown in bold. GT: ground truth, AF: acceleration factor

Parameter	Number of multi-contrast images	(A) qDC-CNN	(B) MANTIS
S0	4	**1.77** ± 0.25	9.09 ± 0.66
	8	**1.67** ± 0.26	7.82 ± 0.72
	16	**1.63** ± 0.26	6.87 ± 0.35
T2	4	**3.03** ± 0.41	19.4 ± 1.18
	8	**2.56** ± 0.36	12.5 ± 0.65
	16	**2.25** ± 0.40	11.6 ± 0.62

## Discussion

### Summary of this study

In this study, we proposed qDC-CNN to accelerate quantitative parameter mapping. qDC-CNN is the E2E framework comprising two networks for image reconstruction and quantitative parameter estimation. We performed the two experiments and showed that qDC-CNN outperforms the other reconstruction methods. Indeed, the reconstruction errors of T2 for qDC-CNN were much smaller than for the conventional method MANTIS. The performance of the reconstructed S0 image was also very high compared to other methods, as was the T2 image. The evaluation of NMRSE values across different models in Experiment 1 showed the proposed model improved performance using data consistency and parameter mapping losses and by using FCNN rather than LSF. Previous studies [21] reported that FCNN is effective in reducing errors, especially for complex signal models. Data consistency and parameter mapping have been shown to be effective in MANTIS, and in this study, it has been shown to be even more successful in unrolled networks using FCNNs. Therefore, qDC-CNN also has potential applications for complicated signal models that do not have analytical solutions.

### Limitations

Several limitations exist in our study. First, we only used a single-coil dataset to simplify the study design. Multi-receiver coils are widely used in clinical MRI, and multi-coil datasets may allow for larger AF than single-coil data sets [30]. The proposed qDC-CNN can also be extended to multi-coil data by modifying the DC-CNN of the image reconstruction network [31, 32].

The second limitation is the use of simulation data only. The data acquired on an accurate scanner may differ from the simulation data with respect to two aspects. First, actual clinical images often suffer from artifacts caused by hardware imperfections, including main field, gradient field, and radiofrequency field variations. If the training data changes and domain shifts occur, the performance of the proposed model is expected to deteriorate, as in the case of many DL reconstruction models [31]. However, in clinical examinations, pulse sequences and hardware configurations are optimized and adjusted so that these artifacts are negligibly small. Therefore, there seems to be little impact on the performance of the proposed method, and if the artifacts are severe, the proposed method would work well if trained using data acquired in actual clinical practice. Second, the simple signal model used in this study would differ from actual signal evolution, including stimulated and indirect echoes. The same simple model was used in the MANTIS model, which has been to guarantee the same performance on in vivo data sets as on simulation data sets, and the same would be true for the proposed method. For applications in low-field MRI or other sequences with significant hardware effects, the framework can be modified to use simulation-based numerical solutions, such as EPG [33, 34] or Bloch simulation [35], instead of analytical solutions. This adaptation would allow the creation of a more comprehensive dictionary-based signal model that better reflects real-world scanning conditions. In the future, validation with in vivo data acquired on a clinical machine is necessary for clinical application. SKM-TEA [36], a multi-coil dataset acquired with the qDESS sequence, and OAI (https://data-archive.nimh.nih.gov/oai/), a single-coil dataset acquired with the MSME sequence, have been published as qMRI, and studies using these datasets will be necessary for the future.

A third limitation is the use of brain data only. Thus, there is an open question as to how much better the performance of the proposed approach is compared to other approaches when it is applied to different anatomical regions. The unrolled network and FCNN have been reported to be useful for the knee and heart [17, 18, 22], and the proposed method is also likely to perform well in these regions. Future work will investigate the effectiveness of the proposed method for other anatomical structures.

### Comparison with previous studies

We compared the proposed method with the baseline method for qMRI reconstruction, MANTIS [23]. In contrast to qDC-CNN, MANTIS showed artifacts and excessive smoothing in the estimated parameter maps, even for the smallest AF of 5. This is inconsistent with the previous study [23], which shows high reconstruction performance for AF = 8. MANTIS uses the data-driven U-Net. The previous study showed that a data-driven network like MANTIS requires more training data than model-based networks to achieve equivalent performance [31]. The number of training images in the present study was small (960 images), which may be the reason for the measured low performance of MANTIS. Another reason for the observed discrepancy may be the difference in the targeted anatomical structures. The knee images were targeted in the previous study, while the more complicated brain images were tested in this study. The previous study [31] reported that U-Net-based models may not have reproduced complicated features.

Recently, another qMRI reconstruction DNN, DOPAMINE [25], which uses an unrolled network, has also been proposed. The main difference between DOPAMINE and our qDC-CNN lies in how the signal model is incorporated into the network. In DOPAMINE, the network is constructed by formulating a DL-constrained convex optimization problem involving the signal model and expanding it using CG methods. In qDC-CNN, on the other hand, the signal model is incorporated into the loss function. Therefore, qDC-CNN can be applied to qMRI sequences for which the signal model is not analytically formulated. For example, 3D-QALAS [10] uses a signal evolution dictionary for parameter mapping, simulated by the Bloch equation [37] or an EPG [33], because the signal model is not provided in a form differentiable by quantitative values. Such qMRI sequences can only be formulated by qDC-CNNs.

### Findings on model structure

Compared to DC-CNN without the mapping module, qDC-CNN showed superior results. Two factors could be responsible for this. First, using FCNNs instead of LSFs in qDC-CNN may have resulted in a more stable quantitative estimation. The second reason is the effect of joint training on both the image reconstruction and quantitative estimation networks. If both networks were trained separately, the reconstruction error of the first image reconstruction network would propagate to the quantitative estimation network in the next step. In contrast, since qDC-CNN trained the weights of the entire network at once through joint training, the final output error could be effectively reduced, leading to the optimized quantitative estimation depending on the sampling conditions.

In addition, we proposed to use pixel-wise FCNN for the mapping module. Instead of pixel-wise, a patch-wise processing network, such as the U-Net used in MANTIS [23], can also be used as a parameter mapping network. This type of network can add surrounding spatial information to the calculation of quantitative values, so it can help improve the accuracy of estimated quantitative maps. On the other hand, there is a risk that the performance of these networks will deteriorate if the anatomical structures of the training and test data differ (U-Net is particularly susceptible to this “domain shift” [31]). On the other hand, pixel-wise networks are robust against performance degradation due to the domain shift.

We pretrained the mapping module before joint E2E network training. Although such pretraining is not always necessary, it has been reported that pretraining improves the stability of training in the case of joint training where multiple networks are combined [38]. We used pretraining because we experienced in the preliminary experiment that the training convergence tends to decrease without pretraining. The time required for this pretraining is only a few hours and does not increase the time and effort needed.

Another network can replace the image reconstruction network in qDC-CNN. In addition to the DC-CNN used in this study, many other methods have been proposed as unrolling networks (e.g., MoDL [16] and variational network [17], which support multi-coil data), and their reconstruction performance differs depending on the reconstruction conditions. The proposed framework has the flexibility to improve the overall performance by selecting the optimal reconstruction module according to the user's requirements.

### Comparison between experiments

In this study, Experiment 1 focused on the performance when AF was increased, while Experiment 2 focused on the performance when the number of contrasts was reduced. For the multi-echo sequence used in this study, the total acquisition time was equal to the acquisition time of the single-contrast image and was not related to the number of multi-contrast images, so the total acquisition time was inversely proportional to the AF. The results suggest that the performance degradation of reconstruction in qDC-CNN is less when the number of multi-contrast images is reduced than when the AF of a single-contrast image is reduced. On the other hand, in the case of other quantitative MRI that does not use simultaneous multi-echo acquisition, such as inversion recovery T1 measurements, or when multiple parameters (e.g., T1rho and T2) are jointly quantified, the acquisition time is determined by both the acquisition time of the single-contrast image and the number of multi-contrast images [24]. Investigating how much speedup can be achieved by increasing AF and reducing the number of multicontrast images remains a future issue.

## Conclusion

In this study, we proposed qDC-CNN as a framework for accelerated quantitative parameter mapping. The proposed method is an E2E framework that combines the two network modules for image reconstruction and quantitative parameter estimation. The performance of the qDC-CNN was evaluated with varying AF and the number of contrast images. The proposed model exhibited smaller NRMSE values than the competing methods, and it had approximately one-quarter of the S0 and T2 NRMSE values compared to MANTIS. The proposed method can be applied to other qMRI sequences and has the flexibility to replace the image reconstruction module to improve performance.

## Supplementary Information

Below is the link to the electronic supplementary material.

Supplementary file1 (DOCX 778 KB)
