# Supplementary material for: Model-based deep learning with fully connected neural networks for accelerated magnetic resonance parameter mapping
Source: Int J Comput Assist Radiol Surg. 2025 May 3;20(12):2437–47. doi: 10.1007/s11548-025-03356-7 (PMC12689753; doi:10.1007/s11548-025-03356-7)
Supplement: Supplementary file 1 — Supplementary file1 (DOCX 778 KB) [file 11548_2025_3356_MOESM1_ESM.docx]

**Supplementary Information**

1. **network implementation**

**A1. Reconstruction Module**

The network structure of the image reconstruction module is shown in Supplementary Figure S1. To address the problem as a multi-contrast image reconstruction problem, we used Equation (2) and formulated it as follows: L2 norm using DNN for the regularization term.

$$\begin{aligned} x_{rec}=\underset{x}{argmin} \left\| x - f_{dnn}(x;\theta) \right\|_{2}^{2}+{\lambda\left\| \boldsymbol{E}_{\boldsymbol{\Omega}}x - y_{\Omega} \right\|}_{2}^{2}\#\left( S1 \right) \end{aligned}$$

Here, $f_{dnn}$ represents the transformation with the DNN parameterized by $\theta$. Equation (S1), without considering noise, can give a closed-form solution with the following equation.

$$\begin{aligned} x_{rec}=E_{U}^{H}E_{\bar{\Omega}}f_{dnn}(x_{0};\theta)+{E_{U}^{H}y}_{\Omega}\#\left( S2 \right) \end{aligned}$$

Here, $U$ represents the entire region of k-space in the whole set, $\Omega$ represents the sampled region, and $\bar{\Omega}$ represents the complement of $\Omega$. Equation (S2) can be rewritten as the following two equations by replacing $f_{dnn}$ with $D_{w}$.

$$\begin{aligned} z=D_{w}\left( x_{0};\theta\right)\#\left( S3 \right) \end{aligned}$$

$$\begin{aligned} x_{rec}=E_{U}^{H}\left( E_{\bar{\Omega}}z+y_{\Omega} \right)\#\left( S4 \right) \end{aligned}$$

Here, $z$ is the intermediate image of the CNN output. $D_{w}\left( x;\theta\right)$ is a denoiser parameterized by $\theta$ with image $x_{0}$ as an input, and is responsible for removing CS-derived noise-like artifact. $E_{U}^{H}\left( E_{\bar{\Omega}}z+y_{\Omega} \right)$ is called the data consistency (DC) layer, in which the sampled regions of the k-space data computed from the denoiser output image are replaced with observed data. Based on equation (S4), the DC-CNN has a cascade structure in which the operations in equations (S3) and (S4) are repeated alternately to increase the expressive power of the network and can be formulated as an incremental formula as follows.

$$\begin{aligned} x_{n+1}=E_{U}^{H}E_{\bar{\Omega}}D_{w}^{n}(x_{n};\theta)+{E_{U}^{H}y}_{\Omega}\#\left( S5 \right) \end{aligned}$$

Here, $D_{w}^{n}(x_{n};\theta)$ is the denoiser at the n-th iteration, and $x_{0}={E_{\Omega}^{H}y}_{\Omega}$. In this study, the number of CNN layers of denoiser, $n_{c}$ was 6, the number of filters, $n_{f}$ was 64, the kernel size was 3×3, and the number of network iterations, $T$ was 5.

**A2. Mapping module**

Quantitative parameter estimation in this study was treated as a pixel-wise operation, similar to LSF.　The input/output of the mapping module,$f_{map}$ is expressed in Equation (6), where the quantitative parameter estimation operation is performed on a per-pixel basis using FCNN. The input of the internal FCNN is the signal intensities $S_{1}(i,j),\ldots,$and $S_{n}(i,j)$ of the multi-contrast images, and the output is the quantitative parameters, $\tilde{S}_{0}\left( i,j \right)$ and $\tilde{T}_{2}(i,j)$. Here, $i,j$ is the index number of the image pixel. The number of dimensions of the input is 2P (P is the number of multi-contrast images) and the number of dimensions of the output was 3, since both multi-contrast images and quantitative parameters are treated as complex values. The number of dimensions of the intermediate layers of the FCNN was 128, and the number of layers was 3 (Supplementary Figure S2 - (a)).

In this study, pre-training was performed to increase the stability of the E2E training; like the procedure performed by Sasaki et al. [2], a signal model was used to simulate $1.0\times{10}^{7}$ pairs of signal series and quantitative value data for randomly generated parameter values, which were used for pre-training (Supplementary Figure S2 - (b)). For the simulations, T2 values ranged from 0 ms to 500 ms, and S0 values ranged from 0.5 to 1.5. The batch size was set to 25600, and MSE was used as the loss function.

1. **Experiments**

**B1. Learning environment**

Python (version 3; Python Software Foundation, Wilmington, DE) was used for the training and testing process. Each DL network was created using the Keras framework [3] with the TensorFlow backend [4]; K-t SLR used MATLAB (version 9.14, R2023a, The MathWorks Inc. California). The hardware configuration was a 64 bit Ubuntu Linux System (Canonical Ltd., London), NVidia GeForce GTX 2080Ti (11 GB RAM) and NVidia GeForce GTX 1080Ti (11 GB RAM) as GPUs.

**B2. Sampling patterns**

The k-space sampling region of each contrast image can be chosen independently (Supplementary figure S4). Liu et al [5] exploited this to extend the random sampling strategy in CS to the undersampling in the contrast space as well. The TE values were 10, 50, 110, and 150 ms for 4 echo acquisition; 10, 30, 50, 70, 90, 110, 130, and 150 ms for 8 echo acquisition; and 10, 20, 30, 40, 50, 60, 70, 80, 90,100, 110, 120, 130, 140, 150, and 160 ms for 16 echo acquisition. The sampling patterns are shown in Supplementary figure S5.

1. **Impact of lambda parameters**

The results of the investigation of the impact of the hyperparameters λp and λdc on the qDC-CNN proposed in this study are described in this section. To investigate the impact of λp, λdc was fixed at 0 and λp was varied from 0, 0.01, 0.1, 1, 10, and 100. Similarly, to investigate the effect of λdc, we fixed λp at 0.01 and varied λdc as 0, 1e-6, 1e-3, and 1. The numerical evaluation of the reconstructed S0 and T2 images is given in Supplementary Table S3, and the numerical evaluation of each reconstructed contrast image is given in Supplementary Table S4. The results of the reconstructed S0, T2 images show that the NRMSE values did not show much variation in all parameters. On the other hand, looking at the results of each reconstructed contrast image, there was a significant decrease in performance as λp and λdc were increased. However, the results of the reconstructed images were slightly improved when compared to the case where λp and λdc were set to 0.

**Reference**

1. Schlemper J, Caballero J, Hajnal JV, Price AN, Rueckert D (2017) A deep cascade of convolutional neural networks for dynamic MR image reconstruction. IEEE transactions on Medical Imaging 37:491–503

2. Sasaki K, Masutani Y, Kinoshita K, Nonaka H, Hirokawa Y (2022) Evaluation of Diffusional Kurtosis Inference Using Synthetic q-space Learning and Bias Correction. Japanese Journal of Radiological Technology 78:. https://doi.org/10.6009/jjrt.2022-1214

3. Chollet F, others (2015) Keras. https://github.com/fchollet/keras

4. Martín Abadi, Ashish Agarwal, Paul Barham, Eugene Brevdo, Zhifeng Chen, Craig Citro, Greg S. Corrado, Andy Davis, Jeffrey Dean, Matthieu Devin, Sanjay Ghemawat, Ian Goodfellow, Andrew Harp, Geoffrey Irving, Michael Isard, Jia Y, Rafal Jozefowicz, Lukasz Kaiser, Manjunath Kudlur, Josh Levenberg, Dan Mané, Rajat Monga, Sherry Moore, Derek Murray, Chris Olah, Mike Schuster, Jonathon Shlens, Benoit Steiner, Ilya Sutskever, Kunal Talwar, Paul Tucker, Vincent Vanhoucke, Vijay Vasudevan, Fernanda Viégas, Oriol Vinyals, Pete Warden, Martin Wattenberg, Martin Wicke, Yuan Yu, Xiaoqiang Zheng (2015) TensorFlow: Large-Scale Machine Learning on Heterogeneous Systems

5. Liu F, Feng L, Kijowski R (2019) MANTIS: Model-Augmented Neural neTwork with Incoherent k-space Sampling for efficient MR parameter mapping. Magnetic resonance in medicine 82:174–188


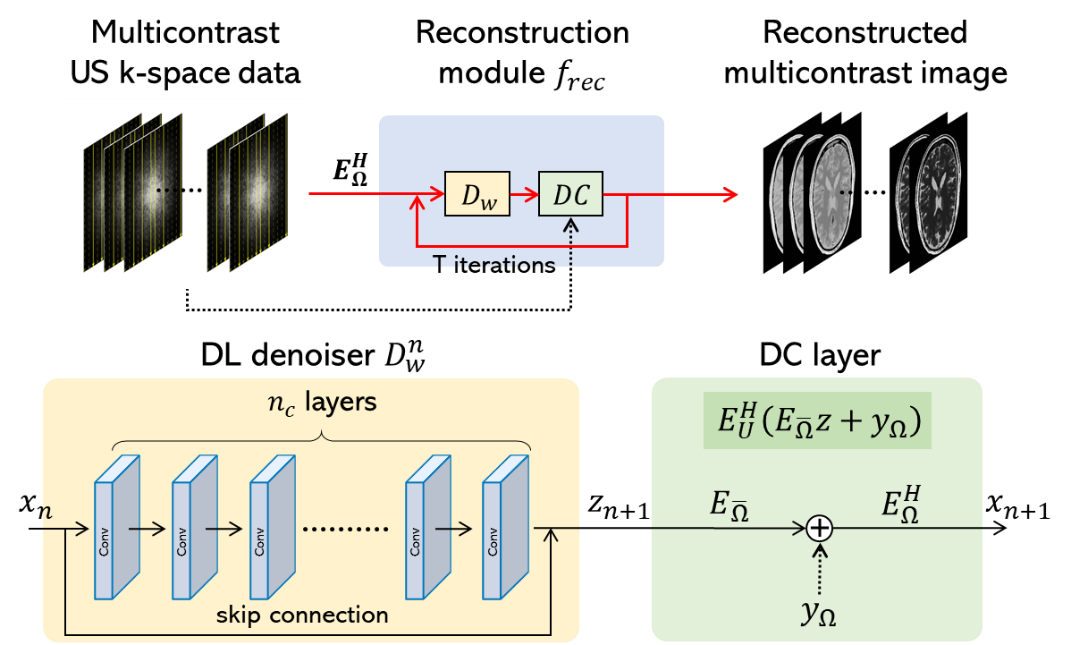
**
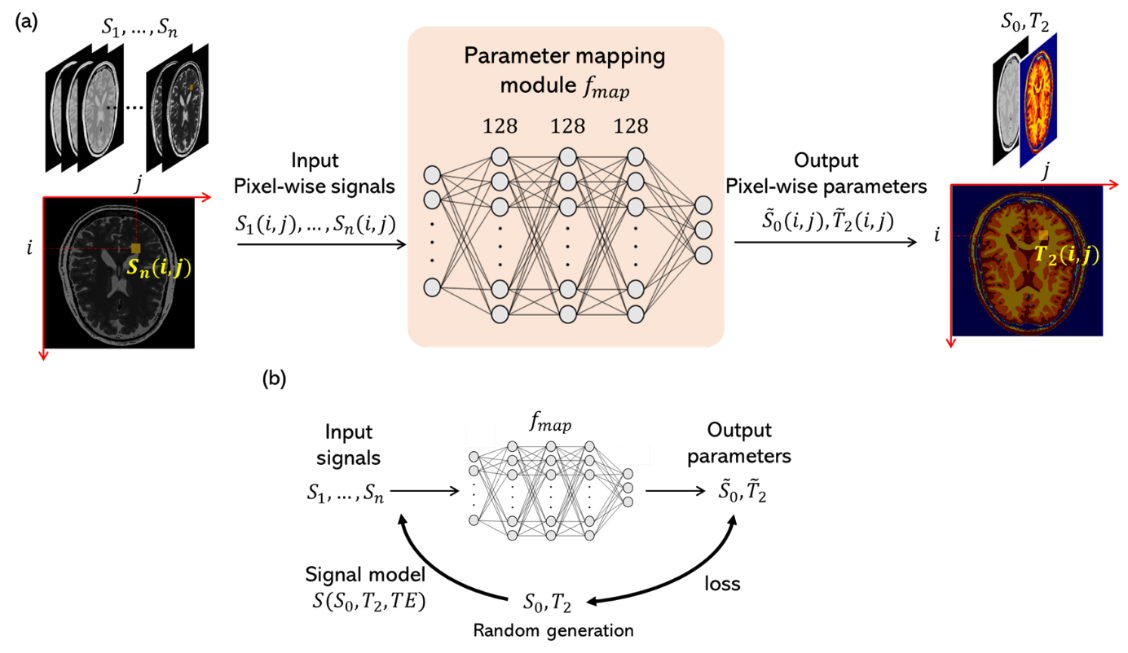
**

**Supplementary Figure S2.** Structure of the mapping module and pre-training procedure. (a) Structure of the network, where $\tilde{S}_{0}(i,j)$ and $\tilde{T}_{2}(i,j)$ are the values at the i-th and j-th pixel index numbers, respectively. (b) Pre-training procedure.

**Supplementary Figure S1.** Input/output and structure of the image reconstruction module. US: Undersampled, DC: data-consistency


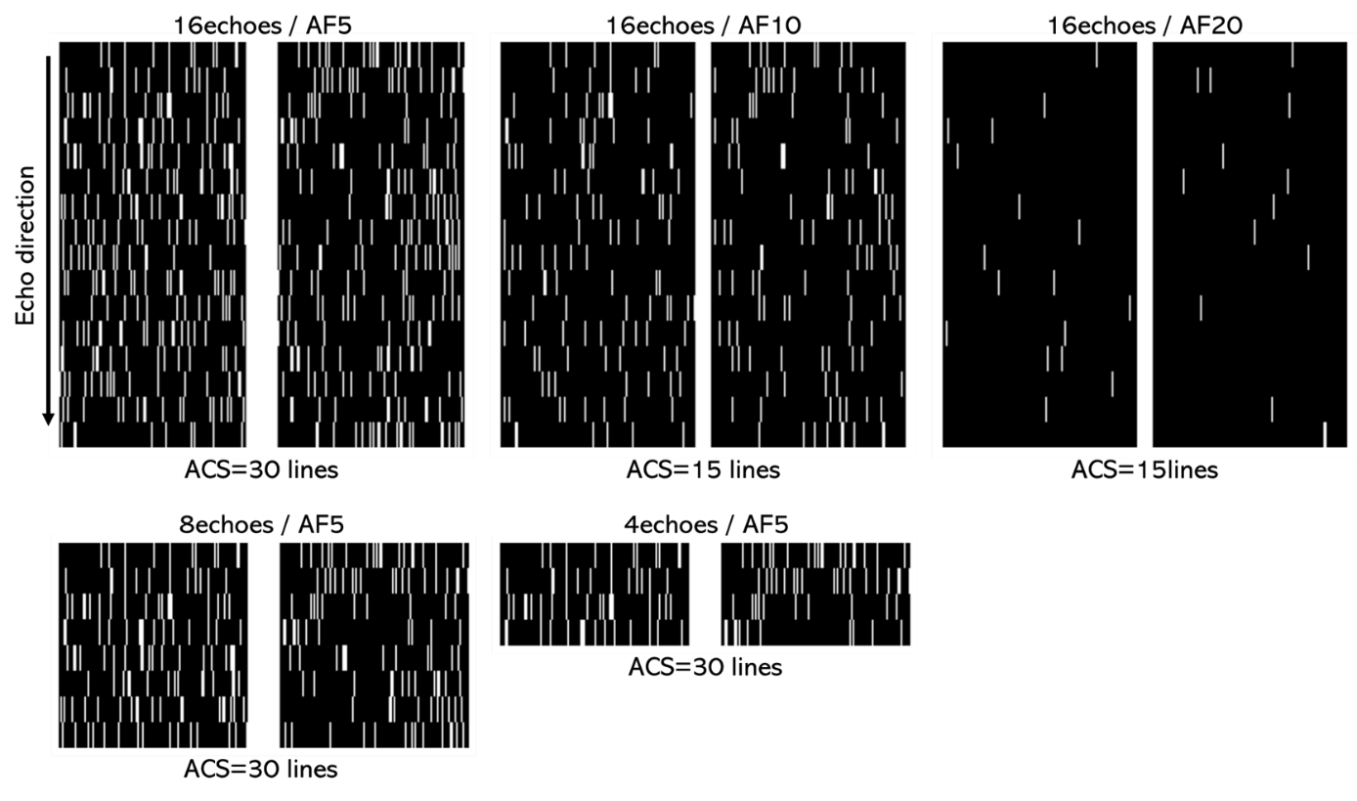

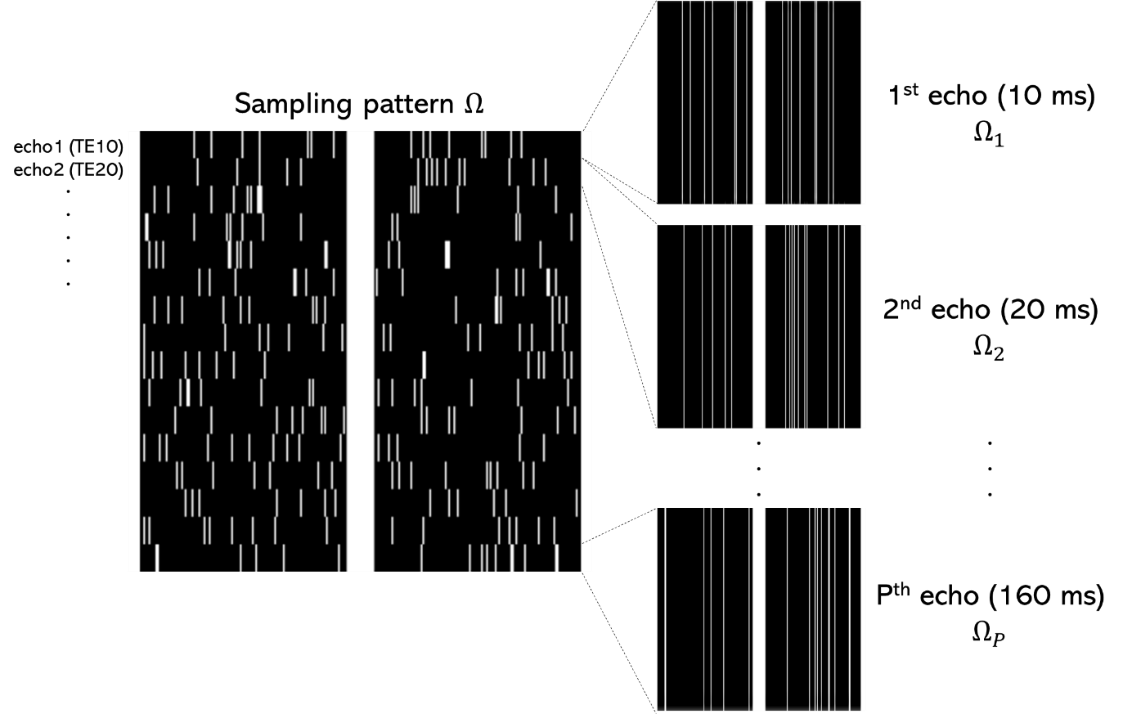


**Supplementary figure S4.** Sampling patterns used in Experiments 1 and 2. AF: acceleration factor, ACS: auto calibration signal.

**Supplementary figure S3.** Examples of sampling patterns for different sampling regions for each contrast k-space. White areas in the image indicate sampled regions, and black areas indicate unsampled regions. The image on the right is the original sampling area for each contrast, and the image on the left shows the sampling areas for each contrast stacked vertically.

Supplementary Table S1. PSNR values to reconstructed images in Experiment 1.

| Contrast | Model | AF5 | AF10 | AF20 |
| --- | --- | --- | --- | --- |
| TE = 10 ms | qDC-CNN | **39.89** ± 0.30 | **36.44** ± 0.20 | **28.56** ± 0.77 |
|  | DC-CNN | 38.81 ± 0.44 | 35.37 ± 0.31 | 27.11 ± 0.61 |
|  | k-t SLR | 29.68 ± 0.43 | 18.43 ± 0.09 | 15.07 ± 0.09 |
|  | ZF | 17.24 ± 0.30 | 14.64 ± 0.09 | 14.60 ± 0.09 |
| TE = 20 ms | qDC-CNN | **40.31** ± 0.35 | **37.33** ± 0.19 | **28.78** ± 0.63 |
|  | DC-CNN | 39.40 ± 0.45 | 36.28 ± 0.27 | 27.23 ± 0.51 |
|  | k-t SLR | 30.61 ± 0.43 | 19.24 ± 0.11 | 15.13 ± 0.09 |
|  | ZF | 16.90 ± 0.29 | 14.90 ± 0.10 | 14.69 ± 0.09 |
| TE = 30 ms | qDC-CNN | **40.40** ± 0.32 | **37.74** ± 0.18 | **28.74** ± 0.53 |
|  | DC-CNN | 39.77 ± 0.43 | 36.62 ± 0.22 | 27.12 ± 0.40 |
|  | k-t SLR | 31.10 ± 0.43 | 19.80 ± 0.13 | 15.07 ± 0.09 |
|  | ZF | 17.02 ± 0.30 | 14.96 ± 0.09 | 14.66 ± 0.10 |
| TE = 40 ms | qDC-CNN | **40.34** ± 0.24 | **37.85** ± 0.18 | **28.65** ± 0.44 |
|  | DC-CNN | 39.88 ± 0.32 | 36.73 ± 0.18 | 26.92 ± 0.34 |
|  | k-t SLR | 31.41 ± 0.42 | 20.12 ± 0.16 | 14.99 ± 0.10 |
|  | ZF | 17.41 ± 0.30 | 14.87 ± 0.13 | 14.61 ± 0.11 |
| TE = 50 ms | qDC-CNN | **40.30** ± 0.22 | **37.85** ± 0.19 | **28.57** ± 0.38 |
|  | DC-CNN | 39.94 ± 0.31 | 36.75 ± 0.16 | 26.79 ± 0.30 |
|  | k-t SLR | 31.31 ± 0.41 | 20.40 ± 0.19 | 14.94 ± 0.11 |
|  | ZF | 16.93 ± 0.29 | 14.80 ± 0.13 | 14.57 ± 0.12 |
| TE = 60 ms | qDC-CNN | **40.02** ± 0.18 | **37.61** ± 0.20 | **28.36** ± 0.35 |
|  | DC-CNN | 39.66 ± 0.27 | 36.59 ± 0.14 | 26.51 ± 0.28 |
|  | k-t SLR | 31.05 ± 0.40 | 20.46 ± 0.22 | 14.80 ± 0.12 |
|  | ZF | 16.58 ± 0.29 | 14.58 ± 0.14 | 14.45 ± 0.12 |
| TE = 70 ms | qDC-CNN | **39.70** ± 0.22 | **37.42** ± 0.19 | **28.13** ± 0.34 |
|  | DC-CNN | 39.41 ± 0.26 | 36.34 ± 0.12 | 26.20 ± 0.28 |
|  | k-t SLR | 30.81 ± 0.38 | 20.55 ± 0.24 | 14.61 ± 0.14 |
|  | ZF | 16.42 ± 0.31 | 14.39 ± 0.14 | 14.23 ± 0.14 |
| TE = 80 ms | qDC-CNN | **39.55** ± 0.17 | **37.37** ± 0.17 | **28.00** ± 0.33 |
|  | DC-CNN | 39.34 ± 0.17 | 36.30 ± 0.11 | 26.08 ± 0.28 |
|  | k-t SLR | 30.52 ± 0.38 | 20.65 ± 0.27 | 14.54 ± 0.15 |
|  | ZF | 16.34 ± 0.29 | 14.51 ± 0.19 | 14.17 ± 0.15 |
| TE = 90 ms | qDC-CNN | **39.34** ± 0.18 | **37.00** ± 0.19 | **27.79** ± 0.33 |
|  | DC-CNN | 38.87 ± 0.24 | 36.03 ± 0.10 | 25.81 ± 0.29 |
|  | k-t SLR | 30.28 ± 0.36 | 20.62 ± 0.29 | 14.38 ± 0.17 |
|  | ZF | 15.89 ± 0.30 | 14.00 ± 0.17 | 13.99 ± 0.16 |
| TE = 100 ms | qDC-CNN | **39.14** ± 0.13 | **36.92** ± 0.17 | **27.66** ± 0.34 |
|  | DC-CNN | 38.83 ± 0.18 | 35.96 ± 0.11 | 25.63 ± 0.30 |
|  | k-t SLR | 30.22 ± 0.35 | 20.68 ± 0.31 | 14.29 ± 0.18 |
|  | ZF | 16.07 ± 0.31 | 14.19 ± 0.18 | 13.88 ± 0.17 |
| TE = 110 ms | qDC-CNN | **39.00** ± 0.08 | **36.83** ± 0.15 | **27.55** ± 0.34 |
|  | DC-CNN | 38.68 ± 0.11 | 35.86 ± 0.10 | 25.56 ± 0.30 |
|  | k-t SLR | 29.96 ± 0.34 | 20.67 ± 0.34 | 14.22 ± 0.19 |
|  | ZF | 16.08 ± 0.31 | 14.24 ± 0.17 | 13.88 ± 0.19 |
| TE = 120 ms | qDC-CNN | **38.75** ± 0.08 | **36.69** ± 0.14 | **27.43** ± 0.35 |
|  | DC-CNN | 38.44 ± 0.12 | 35.70 ± 0.10 | 25.40 ± 0.32 |
|  | k-t SLR | 29.88 ± 0.33 | 20.62 ± 0.39 | 14.16 ± 0.20 |
|  | ZF | 15.75 ± 0.30 | 14.09 ± 0.23 | 13.75 ± 0.19 |
| TE = 130 ms | qDC-CNN | **38.65** ± 0.07 | **36.44** ± 0.14 | **27.25** ± 0.36 |
|  | DC-CNN | 38.33 ± 0.10 | 35.51 ± 0.11 | 25.20 ± 0.32 |
|  | k-t SLR | 29.69 ± 0.31 | 20.41 ± 0.43 | 14.01 ± 0.20 |
|  | ZF | 15.83 ± 0.31 | 13.91 ± 0.21 | 13.61 ± 0.20 |
| TE = 140 ms | qDC-CNN | **38.35** ± 0.09 | **36.17** ± 0.13 | **27.15** ± 0.36 |
|  | DC-CNN | 38.00 ± 0.08 | 35.28 ± 0.11 | 25.09 ± 0.33 |
|  | k-t SLR | 29.61 ± 0.31 | 20.14 ± 0.44 | 13.91 ± 0.21 |
|  | ZF | 15.63 ± 0.32 | 13.69 ± 0.21 | 13.57 ± 0.20 |
| TE = 150 ms | qDC-CNN | **38.20** ± 0.09 | **36.03** ± 0.12 | **26.99** ± 0.37 |
|  | DC-CNN | 37.88 ± 0.08 | 35.16 ± 0.11 | 24.91 ± 0.33 |
|  | k-t SLR | 29.50 ± 0.29 | 19.90 ± 0.46 | 13.78 ± 0.21 |
|  | ZF | 15.70 ± 0.31 | 13.56 ± 0.22 | 13.42 ± 0.21 |
| TE = 160 ms | qDC-CNN | **37.93** ± 0.07 | **35.87** ± 0.13 | **26.90** ± 0.38 |
|  | DC-CNN | 37.39 ± 0.11 | 34.94 ± 0.11 | 24.79 ± 0.34 |
|  | k-t SLR | 29.46 ± 0.27 | 19.64 ± 0.46 | 13.71 ± 0.22 |
|  | ZF | 15.17 ± 0.32 | 13.48 ± 0.23 | 13.36 ± 0.21 |

Supplementary Table S2. PSNR values to reconstructed images in Experiment 2.

| Contrast | P = 4 | P = 8 | P = 16 |
| --- | --- | --- | --- |
| TE = 10 ms | 39.20 ± 0.37 | 39.56 ± 0.43 | **39.89** ± 0.30 |
| TE = 20 ms | - | - | 40.31 ± 0.35 |
| TE = 30 ms | - | 40.09 ± 0.30 | **40.40** ± 0.32 |
| TE = 40 ms | - | - | 40.34 ± 0.24 |
| TE = 50 ms | - | 39.96 ± 0.19 | **40.30** ± 0.22 |
| TE = 60 ms | 38.82 ± 0.23 | - | **40.02** ± 0.18 |
| TE = 70 ms | - | 39.52 ± 0.12 | **39.70** ± 0.22 |
| TE = 80 ms | - | - | 39.55 ± 0.17 |
| TE = 90 ms | - | **39.34** ± 0.10 | **39.34** ± 0.18 |
| TE = 100 ms | - | - | 39.14 ± 0.13 |
| TE = 110 ms | 37.96 ± 0.12 | 38.77 ± 0.09 | **39.00** ± 0.08 |
| TE = 120 ms | - | - | 38.75 ± 0.08 |
| TE = 130 ms | - | 38.49 ± 0.11 | **38.65** ± 0.07 |
| TE = 140 ms | - | - | 38.35 ± 0.09 |
| TE = 150 ms | - | 37.95 ± 0.10 | **38.20** ± 0.09 |
| TE = 160 ms | 37.09 ± 0.11 | - | **37.93** ± 0.07 |

Supplementary Table S3. NRMSE values in reconstructed S0/T2 images in variation of lambda values

| Parameter | Term |  | NRMSE |
| --- | --- | --- | --- |
| S0 | $\lambda_{p}$ | $1.0\times{10}^{-2}$ | **3.05** ± 0.14 |
|  |  | $1.0\times{10}^{-1}$ | 3.12 ± 0.19 |
|  |  | $1.0$ | 3.44 ± 0.17 |
|  |  | $1.0\times{10}^{1}$ | 3.57 ± 0.22 |
|  |  | $1.0\times{10}^{2}$ | 3.39 ± 0.19 |
|  | $\lambda_{dc}$ | $0$ | **3.05** ± 0.14 |
|  |  | $1.0\times{10}^{-6}$ | 3.09 ± 0.15 |
|  |  | $1.0\times{10}^{-3}$ | 5.35 ± 0.22 |
|  |  | $1.0$ | 5.89 ± 0.19 |
| T2 | $\lambda_{p}$ | $1.0\times{10}^{-2}$ | 3.92 ± 0.14 |
|  |  | $1.0\times{10}^{-1}$ | 3.99 ± 0.14 |
|  |  | $1.0$ | **3.79** ± 0.17 |
|  |  | $1.0\times{10}^{1}$ | 4.16 ± 0.18 |
|  |  | $1.0\times{10}^{2}$ | 4.20 ± 0.14 |
|  | $\lambda_{dc}$ | $0$ | **3.92** ± 0.14 |
|  |  | $1.0\times{10}^{-6}$ | 4.08 ± 0.21 |
|  |  | $1.0\times{10}^{-3}$ | 5.98 ± 0.28 |
|  |  | $1.0$ | 7.26 ± 0.32 |

Supplementary Table S4. NRMSE values in reconstructed images in variation of lambda values

|  | $\lambda_{p}$ | | | | | |
| --- | --- | --- | --- | --- | --- | --- |
| Contrast | $0$ | $1.0\times{10}^{-2}$ | $1.0\times{10}^{-1}$ | $1.0$ | $1.0\times{10}^{1}$ | $1.0\times{10}^{2}$ |
| TE = 10 ms | 35.37 ± 0.31 | **35.79** ± 0.20 | 35.10 ± 0.26 | 33.49 ± 0.18 | 32.06 ± 0.24 | 29.68 ± 0.15 |
| TE = 20 ms | 36.28 ± 0.27 | **36.78** ± 0.19 | 35.80 ± 0.18 | 33.87 ± 0.17 | 29.32 ± 0.24 | 24.39 ± 0.15 |
| TE = 30 ms | **36.62** ± 0.22 | 36.42 ± 0.24 | 36.12 ± 0.15 | 33.78 ± 0.14 | 30.78 ± 0.14 | 24.81 ± 0.09 |
| TE = 40 ms | **36.73** ± 0.18 | 36.68 ± 0.27 | 36.22 ± 0.15 | 34.04 ± 0.16 | 31.34 ± 0.20 | 30.04 ± 0.10 |
| TE = 50 ms | 36.75 ± 0.16 | **37.14** ± 0.26 | 36.15 ± 0.16 | 34.07 ± 0.19 | 30.90 ± 0.20 | 27.23 ± 0.15 |
| TE = 60 ms | 36.59 ± 0.14 | **36.78** ± 0.29 | 36.03 ± 0.21 | 33.54 ± 0.18 | 31.03 ± 0.18 | 29.01 ± 0.13 |
| TE = 70 ms | 36.34 ± 0.12 | **36.65** ± 0.28 | 35.96 ± 0.19 | 33.44 ± 0.19 | 31.41 ± 0.15 | 27.11 ± 0.13 |
| TE = 80 ms | 36.30 ± 0.11 | **36.53** ± 0.29 | 35.98 ± 0.18 | 33.86 ± 0.17 | 31.16 ± 0.15 | 27.64 ± 0.14 |
| TE = 90 ms | **36.03** ± 0.10 | 35.95 ± 0.29 | 35.69 ± 0.18 | 33.76 ± 0.19 | 30.97 ± 0.16 | 26.15 ± 0.15 |
| TE = 100 ms | 35.96 ± 0.11 | **36.31** ± 0.23 | 35.71 ± 0.18 | 33.82 ± 0.16 | 31.47 ± 0.13 | 29.64 ± 0.11 |
| TE = 110 ms | 35.86 ± 0.10 | **36.11** ± 0.23 | 35.71 ± 0.14 | 33.72 ± 0.15 | 30.61 ± 0.12 | 29.11 ± 0.10 |
| TE = 120 ms | 35.70 ± 0.10 | **36.00** ± 0.24 | 35.54 ± 0.14 | 33.75 ± 0.16 | 30.86 ± 0.12 | 27.99 ± 0.10 |
| TE = 130 ms | 35.51 ± 0.11 | **35.62** ± 0.25 | 35.41 ± 0.15 | 33.31 ± 0.17 | 30.76 ± 0.16 | 26.05 ± 0.12 |
| TE = 140 ms | 35.28 ± 0.11 | **35.42** ± 0.24 | 35.20 ± 0.14 | 33.23 ± 0.13 | 30.65 ± 0.09 | 27.50 ± 0.11 |
| TE = 150 ms | **35.16** ± 0.11 | 34.42 ± 0.28 | 35.14 ± 0.15 | 33.19 ± 0.14 | 30.28 ± 0.10 | 27.66 ± 0.07 |
| TE = 160 ms | 34.94 ± 0.11 | **34.99** ± 0.26 | 34.93 ± 0.13 | 32.29 ± 0.12 | 30.64 ± 0.08 | 24.32 ± 0.10 |

|  | $\lambda_{dc}$ | | | |
| --- | --- | --- | --- | --- |
| Contrast | $0$ | $1.0\times{10}^{-6}$ | $1.0\times{10}^{-3}$ | $1.0$ |
| TE = 10 ms | **35.79** ± 0.20 | 35.57 ± 0.27 | 32.36 ± 0.28 | 31.13 ± 0.27 |
| TE = 20 ms | **36.78** ± 0.19 | 36.63 ± 0.22 | 31.31 ± 0.19 | 28.02 ± 0.26 |
| TE = 30 ms | 36.42 ± 0.24 | **36.81** ± 0.19 | 33.13 ± 0.15 | 27.62 ± 0.29 |
| TE = 40 ms | 36.68 ± 0.27 | **37.07** ± 0.16 | 33.74 ± 0.16 | 32.72 ± 0.20 |
| TE = 50 ms | 37.14 ± 0.26 | **37.16** ± 0.16 | 30.63 ± 0.14 | 28.84 ± 0.30 |
| TE = 60 ms | 36.78 ± 0.29 | **36.80** ± 0.17 | 33.12 ± 0.20 | 29.39 ± 0.31 |
| TE = 70 ms | 36.65 ± 0.28 | **36.74** ± 0.16 | 33.92 ± 0.20 | 32.41 ± 0.44 |
| TE = 80 ms | 36.53 ± 0.29 | **36.61** ± 0.14 | 33.37 ± 0.18 | 31.31 ± 0.35 |
| TE = 90 ms | 35.95 ± 0.29 | **36.36** ± 0.16 | 33.02 ± 0.19 | 29.57 ± 0.50 |
| TE = 100 ms | **36.31** ± 0.23 | 36.30 ± 0.14 | 33.55 ± 0.22 | 31.16 ± 0.35 |
| TE = 110 ms | 36.11 ± 0.23 | **36.21** ± 0.14 | 32.69 ± 0.17 | 29.92 ± 0.41 |
| TE = 120 ms | 36.00 ± 0.24 | **36.12** ± 0.12 | 33.79 ± 0.14 | 30.43 ± 0.36 |
| TE = 130 ms | 35.62 ± 0.25 | **35.93** ± 0.12 | 32.56 ± 0.15 | 29.86 ± 0.37 |
| TE = 140 ms | 35.42 ± 0.24 | **35.68** ± 0.13 | 32.56 ± 0.15 | 31.20 ± 0.39 |
| TE = 150 ms | 34.42 ± 0.28 | **35.36** ± 0.11 | 32.96 ± 0.13 | 30.00 ± 0.44 |
| TE = 160 ms | 34.99 ± 0.26 | **35.38** ± 0.13 | 32.26 ± 0.13 | 29.77 ± 0.30 |
